# Micro-LEGO for MEMS

**DOI:** 10.3390/mi10040267

**Published:** 2019-04-21

**Authors:** Seok Kim

**Affiliations:** Department of Mechanical Science and Engineering, University of Illinois at Urbana-Champaign, 1206 West Green Street, Urbana, IL 61801, USA; skm@illinois.edu; Tel.: +1-217-265-5656

**Keywords:** microassembly, MEMS, transfer printing, elastomer stamp, shape memory polymer

## Abstract

The recently developed transfer printing-based microassembly called micro-LEGO has been exploited to enable microelectromechanical systems (MEMS) applications which are difficult to achieve using conventional microfabrication. Micro-LEGO involves transfer printing and thermal processing of prefabricated micro/nanoscale materials to assemble structures and devices in a 3D manner without requiring any wet or vacuum processes. Therefore, it complements existing microfabrication and other micro-assembly methods. In this paper, the process components of micro-LEGO, including transfer printing with polymer stamps, material preparation and joining, are summarized. Moreover, recent progress of micro-LEGO within MEMS applications are reviewed by investigating several example devices which are partially or fully assembled via micro-LEGO.

## 1. Introduction

As Moore’s law for digital computation has predicted, electrical integrated circuits (IC) and systems have continuously been miniaturized towards improved size, weight, and power consumption (SWaP) through microfabrication over the past several decades [1]. Simultaneously, nearly all other types of devices and systems have been miniaturized with great opportunities for the performance and cost of these systems by adopting batch fabrication technologies successfully employed from the IC industry. In particular, microelectromechanical systems (MEMS) represent an effort of this evolutionary engineering and have enabled many types of sensors, actuators, and systems to be reduced in size, exploiting microfabrication while often improving device performance [2]. For example, MEMS has miniaturized mechanical switches [3,4,5,6,7], chemical and physical sensors [8,9,10,11,12], display mirrors [13,14,15,16,17,18], and power generators [19,20,21,22]. A typical microfabrication process for MEMS encompasses photolithography to form a masking layer for subsequent processes, physical and chemical deposition to create a target material layer, and dry or wet etching to pattern the deposited layer. These constitutive processes are often repeated until the final device architecture is completed [2]. However, these microfabrication processes, especially conducted on a single substrate, have various restrictions in terms of material and process compatibility, complex geometry, and manufacturing flexibility. For example, materials and structures formed early in the process sequence should survive or be protected from any following process conditions, such as corrosive etching, high temperature, and a high vacuum environment. Moreover, each photolithography usually involves a photoresist spin-coating that requires planar morphology of a surface, so that certain planarization or chemical mechanical polishing is often necessary for each photolithography. While there are other photolithography methods, including photoresist electrodeposition for a surface with extreme morphology, they typically require complex process conditions, such as conductive substrates and charged solutions, compared to simple spin-coating [23]. The final allowed structural geometries are also quite limited or require complex process steps to be obtained. For instance, the common fabrication of a free-standing mechanical member needs the predeposition and etching of a sacrificial layer over which a free-standing member exists, while it can be easily implemented by placing a member in the macroworld. Thus, microfabrication processes to build miniaturized devices generally require careful process sequences.

**Figure 1:**
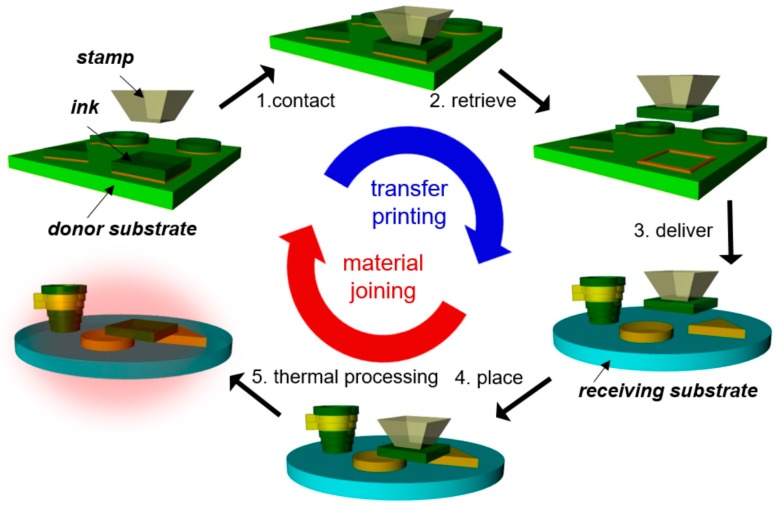
Micro-LEGO procedure, including transfer printing and material joining.

Due to these limiting characteristics of microfabrication, there has been a great need for alternate and complementing approaches, with microassembly being one of those. Since microassembly is relatively material agnostic, different types of materials and devices can be individually optimized, and sequentially or parallelly integrated together, analogous to a macroworld assembly line [24]. Common microassembly technologies are studied roughly in three different domains, including (1) self-assembly relying on energy minimization approaches [25,26], (2) robotic pick-and-place using micromanipulators either in contact or non-contact modes [27,28], and (3) transfer printing-based methods utilizing reversibly adhesive polymer stamps [24]. Approaches in each domain have their advantages and disadvantages regarding material constraints, yield, throughput, and process flexibility. Among these microassembly technologies, transfer printing-based methods have most recently emerged. Transfer printing is a method to transfer ‘inks’, defined here as a diversity of material classes with a wide range of geometries and configurations, from a donor substrate where they are generated or grown to a receiving substrate, where they are then utilized by using controlled dry adhesion of polymer stamps [29,30,31,32,33]. The transfer printing-based microassembly utilizes this transfer printing combined with material joining techniques to assemble microscale heterogeneous solid materials. Hereafter, inks mean exclusively solid-state materials in the context of transfer printing-based microassembly. This unique microassembly technology was first demonstrated with single crystal silicon inks, and thus was named ‘micro-masonry’, due to its structural analogy to traditional masonry [34]. After that, the materials accessible to transfer printing and joining were further extended to silicon dioxide (SiO_2_), gold (Au), and epoxy polymer (SU8) [35]. Therefore, transfer printing-based microassembly was able to accommodate common types of MEMS materials, such as semiconductors, dielectrics, metals, and polymers, and it was renamed ‘micro-LEGO’ to represent its heterogeneous material integration which is illustrated in Figure 1. In this figure, the different colors of inks indicate their different materials. This transfer printing-based microassembly provides several attractive features: (a) functional and structural solid inks of dissimilar materials can be integrated in a 3D manner to assemble MEMS sensors and actuators; (b) the interfaces of assembled solid inks with strong mechanical joining can function as electrical and thermal contacts; (c) the assembly spatial resolution can be high (~1 m) by utilizing scalable photolithographic processes for generating solid inks and precise mechanical stages for transfer printing; (d) functional and structural solid inks can be assembled on both rigid and flexible substrates in planar or curvilinear geometries; and (e) the assembly does not have to be conducted in a cleanroom once solid inks are photolithographically fabricated in densely packed arrangements on donor substrates, resulting in potential resource savings.

In this paper, micro-LEGO which has been developed over the past several years is reviewed with the details of its process components and device applications, particularly enabling 3D MEMS. Following the Introduction, Section 2 of the paper describes soft stamp-based transfer printing and the evolution of stamps through more advanced structural designs, actuation mechanisms, and material selections. Section 3 summarizes methods to prepare inks made of diverse materials, such that they are effectively transfer-printed via stamps. After transfer printing, inks need to be mechanically joined to ensure the structural integrity of final assembled devices. The joining processes between homogenous, as well as heterogeneous materials, are also summarized in Section 4. After reviewing these micro-LEGO process components, representative MEMS device-level applications of micro-LEGO demonstrated thus far are introduced in Section 5. Finally, the future applications and outlook for micro-LEGO are discussed. This paper summarizes currently available process components of micro-LEGO, which have been introduced in different papers mainly related to the author’s prior work, to facilitate broader adoption of micro-LEGO in MEMS and other related fields.

## 2. Transfer Printing

Micro-LEGO has been established as transfer printing—a method to transfer solid materials (i.e., ‘solid inks’) from a donor substrate to a receiving substrate—has evolved. The performance of transfer printing is substantially dictated by the capabilities of stamps, such as their dry adhesion strength and reversibility (maximum to minimum adhesion ratio). In this section, stamps made of two major polymers which are commonly used in micro-LEGO are described.

### 2.1. Elastomer Stamps

**Figure 2:**
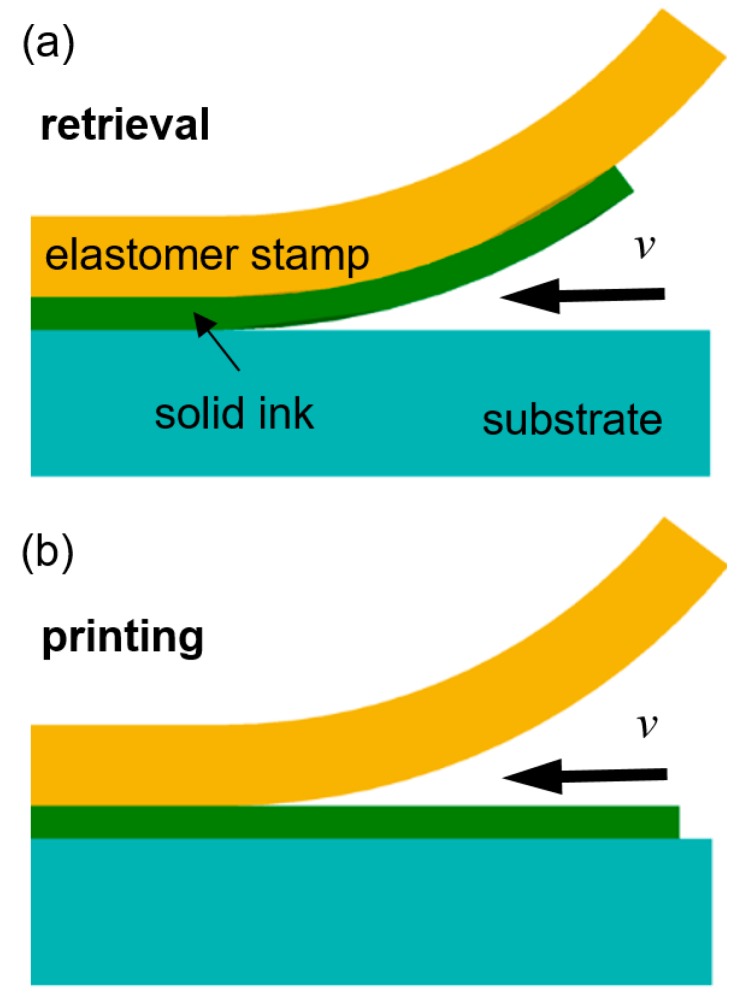
Schematic diagram of retrieval (**a**) and printing (**b**) of a solid ink during transfer printing using an elastomer stamp [36].

In its generic form, transfer printing uses kinetically switchable dry adhesion of elastomer stamps to retrieve solid inks from donor substrates and to deliver and print them on receiver substrates. This transfer printing process can be considered as a competing fracture between two interfaces. As illustrated in Figure 2, when an elastomer stamp is peeled off from a certain surface with steady-state crack propagation at a steady-state speed *v*, the energy release rate *G* is related to the peeling force *F* by [36,37,38,39,40,41]

(1) $$G=\frac{F}{w},$$

where *w* is the width of the stamp. Here, *G* means the energy of interfacial bond breaking as well as viscoelastic energy dissipation near the crack tip. The crack propagates steadily once *G* reaches the critical energy release rate of Griffith criterion in fracture mechanics [42]. The critical energy release rate for the interface between materials A and B is denoted as $G_{crit}^{A/B}$ in this context. is independent of the peeling velocity *v* since typical ink and substrate materials are elastic. However, $G_{crit}^{stamp/ink}$ is a monotonically increasing function of *v* since elastomer stamps are viscoelastic. Thus, $G_{crit}^{stamp/ink}$ is expressed with the below power law [36].

(2) $$G_{crit}^{stamp/ink}\left(\nu \right)=G_{0}\left[1+{\left(\frac{\nu }{\nu_{0}}\right)}^{n}\right],$$

where *G*_0_, *v*_0_, and *n* are constants. Since $G_{crit}^{stamp/ink}\left(\nu \right)$ is changed by tuning the peeling speed *v*, the solid ink can be retrieved ($G_{crit}^{ink/substrate}<G_{crit}^{stamp/ink}\left(\nu \right)$) or printed ($G_{crit}^{ink/substrate}>G_{crit}^{stamp/ink}\left(\nu \right)$) depending on *v*, as depicted in Figure 2 [36]. Transfer printing using this kinetically switchable adhesion has been extensively used to assemble prefabricated solid inks on a variety of target-receiving surfaces with moderate adhesion reversibility (maximum to minimum adhesion ratio of 3–10) [29,30,31].

**Figure 3:**
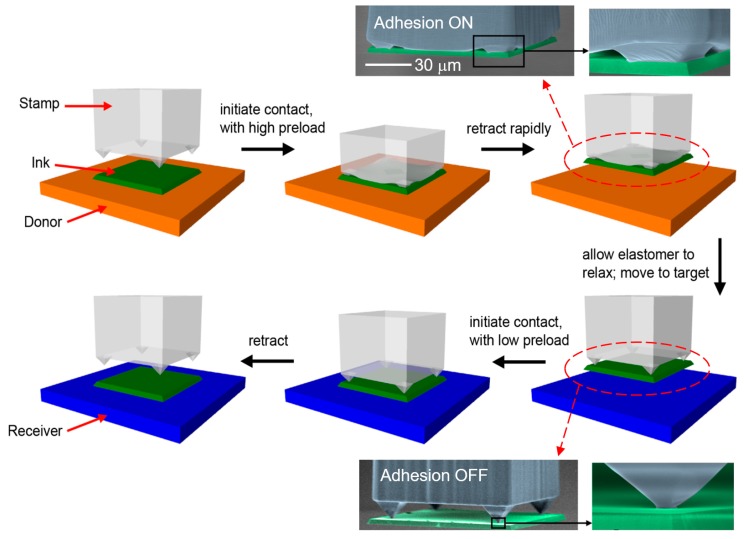
Implementation of an elastomer microtip stamp for transfer printing with corresponding colored SEM images. Reproduced with permission from [31,32].

Over this generic form, several alternate strategies to increase adhesion reversibility have been presented. Surface relief structures (microtips) on a flat stamp surface were designed [31], and an inflatable stamp array with active pressure control was developed [43]. These two types of stamps change their contact area with and control their dry adhesion to an ink upon external mechanical or pneumatic loading. Angled stamps were also exploited, which modulate the total stamp adhesion as a function of external shear loading [44,45,46]. Moreover, a pulsed laser was utilized to initiate stamp–ink separation due to large thermal strain mismatch in between, and thus, to enable zero total stamp adhesion when necessary [47]. Among those, the elastomer stamp with microtips made of polydimethylsiloxane (PDMS) has been the most versatile and commonly utilized stamp in micro-LEGO. This microtip stamp switches its contact area, and, therefore, its adhesion with a solid ink from maximum (ON) to minimum (OFF) by modulating mechanical loading and retraction speed as depicted in Figure 3. The inset colored scanning electron microscope (SEM) images represent a microtip stamp (grey-colored) and a solid ink (green-colored) at adhesion ON and OFF states. Elastomer stamps with this microtip design have enabled fast, convenient, and reliable transfer printing with a high adhesion ON/OFF ratio, i.e., reversibility, and less sensitivity to positioning error which may occur during transfer printing [48].

### 2.2. Shape Memory Polymer Stamps

All of the aforementioned alternate elastomer stamp designs efficiently enhance the adhesion reversibility by decreasing or eliminating the minimum adhesion. However, the maximum adhesion of those elastomer stamps is still kinetically controlled. Accordingly, their maximum adhesion is time sensitive and occurs transiently (<1 s) since it is limited by the viscoelastic properties of elastomers. Stamps with extremely high adhesion reversibility, in addition to time-insensitive and higher maximum adhesion strength, would allow for a more versatile transfer printing process. It is known that a soft material stores greater elastic energy than a stiff material, and therefore, by Griffith criterion [42], the point at which the release of stored elastic energy surpasses the energy required to advance a crack at the adhesive interface occurs at a lower stress. Likewise, the contact for dissimilar stiff materials is less susceptible to peeling failure at the contact interface than the contact between soft and stiff materials, indicating that the maximum adhesion of an elastomer stamp is also limited by the low elastic modulus of an elastomer. For example, the maximum adhesion force of a PDMS stamp, the most common form of elastomer stamps, has been measured to be in the order of atmospheric pressure [49]. Accordingly, using a stamp made of a material with higher elastic modulus evidently increases stamp adhesion. However, if a stamp is stiffer, there is less conformal contact with an opposing surface, resulting in the lower stamp adhesion due to the smaller true contact area in between. To resolve this contradictory condition, that a stamp material must be soft enough to make a conformal contact and increase true contact area but stiff enough to increase the stamp adhesion, shape memory polymers (SMPs) were explored. SMPs are a class of external stimuli-responsive polymers with the ability to memorize a ‘permanent shape’ which is recoverable from a deformed shape, i.e., ‘temporary shape’. In particular, a thermoresponsive SMP undergoes a dramatic change in elastic modulus across the glass transition temperature (*T_g_*) between glassy and rubbery states, and generally shows a strong shape-memory effect, i.e., the ability to stably fix its temporary shape and fully recover its permanent shape [50,51,52]. An SMP is uniquely suited to resolve this contradictory condition since its elastic modulus can be changed dynamically and, therefore, a conformal contact with large true contact area can be formed in the rubbery state and maintained in the glassy state, where its elastic modulus is maximized because of the strong temporary shape-fixing.

**Figure 4:**
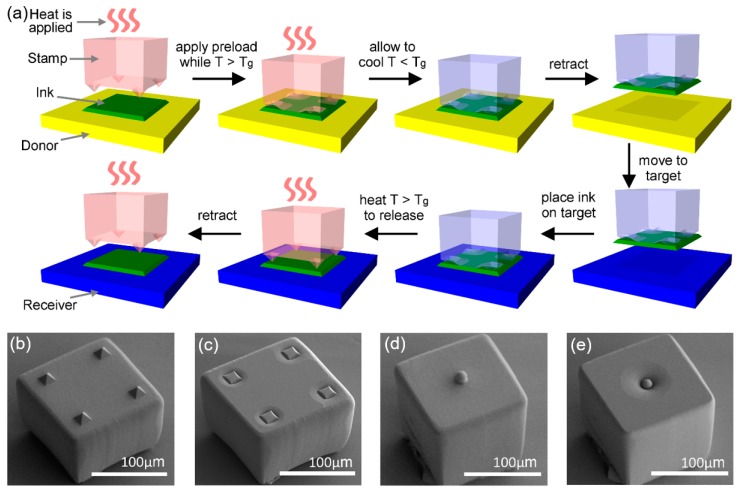
(**a**) Implementation of a shape memory polymer (SMP) microtip stamp for transfer printing. (**b**) Microtip stamp in permanent, “adhesion OFF” state. (**c**) Microtip stamp in temporary “adhesion ON” state. (**d**) Silica-sphere stamp in permanent “adhesion OFF” state. (**e**) Silica-sphere stamp in temporary “adhesion ON” state. Reproduced with permission from [53].

Through exploiting these material properties of SMP, a microtip SMP stamp was designed. Figure 4a illustrates the typical procedure of transfer printing an ink using a microtip SMP stamp [53]. The stamp is shown in Figure 4b is in its ‘permanent’ shape, which is determined by the shape of its mold where it was cured. After temporary shape-fixing, the stamp always returns to this permanent shape, corresponding to its adhesion OFF state where the microtips protrude from the surface to minimize adhesive contact area, when heated. Figure 4c shows the stamp in its ‘temporary’ adhesion ON state, where the microtips have been flattened to allow contact in the interior region of the stamp to maximize the adhesive contact area. As shown in Figure 4a, the adhesion ON state may be maintained throughout the retrieval, delivery, and printing steps, that provide protection from unwanted tilt and rotations which often hinder the printing of complex shape inks when an elastomer stamp is used. An alternate way to further minimize OFF state adhesion to facilitate the printing step was also demonstrated by using an SMP stamp with embedded silica spheres. A silica-sphere SMP stamp is shown in its adhesion OFF state in Figure 4d, and in its adhesion ON state in Figure 4e. The silica sphere is rigid and relatively rough at the submicrometer level, resulting in extremely low adhesion to the ink during the final separation step of printing [54]. Table 1 summarizes the comparison of adhesive strength and reversibility results from several previous publications, to highlight the superior reversible adhesive characteristics of a microtip SMP stamp.

**Figure 5:**
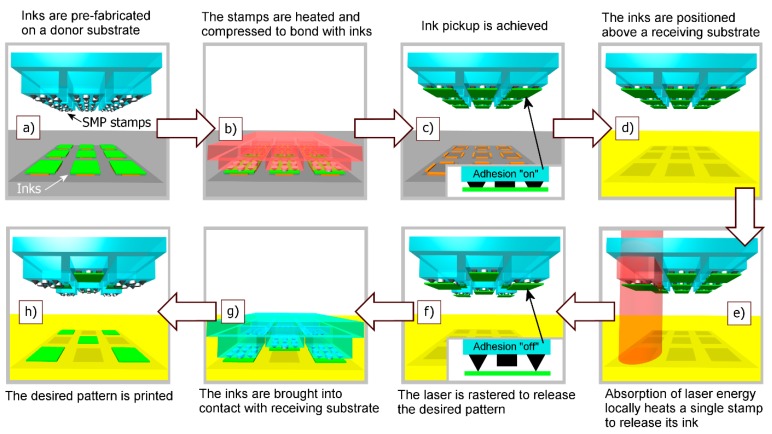
The operation of the laser-driven CBSMP transfer printing process is depicted. Reproduced with permission from [56].

More recently, an SMP stamp array with carbon black composite (CBSMP) microstructuring emerged to enable arbitrary pattern transfer from an array of solid inks via localized control of adhesion [56]. This unique transfer printing approach combines parallelism with individual object control. As shown in Figure 5, heat is first delivered globally by a resistive heater, facilitating parallel ink retrieval. Following this, only selected inks may be precisely printed by local laser illumination absorbed within the CBSMP, while all retrieved inks may also be printed in parallel via the next global heating. In this selective ink printing, the packing density is only limited by the spot size of the laser system that is used. This advanced SMP stamp array has, indeed, resolved one grand challenge of common transfer printing processes that transfer printing works either as a parallel process with high throughput, or as a low throughput process allowing individual manipulation of solid inks. It is worthwhile to note that the batch transfer and assembly of MEMS components using laser-driven release has been previously developed [57]. However, differently from this and other variations, transfer printing exploiting the SMP stamp array with CBSMP microstructuring does not rely on laser ablation of sacrificial materials. A similar approach to controlling individual PDMS stamps combined with parallelism was also demonstrated using an active polymer composite membrane [58].

## 3. Ink Preparation

Before transfer printing for micro-LEGO assembly, materials of interest need to be prepared as inks which are patterned with desired shapes and reliably stay on donor substrates. In addition, their adhesion or joining to donor substrates must be weak enough such that inks are retrieved by stamps without difficulty. Common methods to prepare inks generate patterned inks suspended from a donor substrate using anchors which are strong enough to hold inks but simultaneously weak enough to fail during the ink retrieval by stamps. Up-to-date process protocols to prepare inks from four disparate materials, i.e., Si, SiO_2_, Au, SU8, have been established, but other materials may be explored to prepare as inks through modifying the established protocols which are summarized in this section.

**Figure 6:**
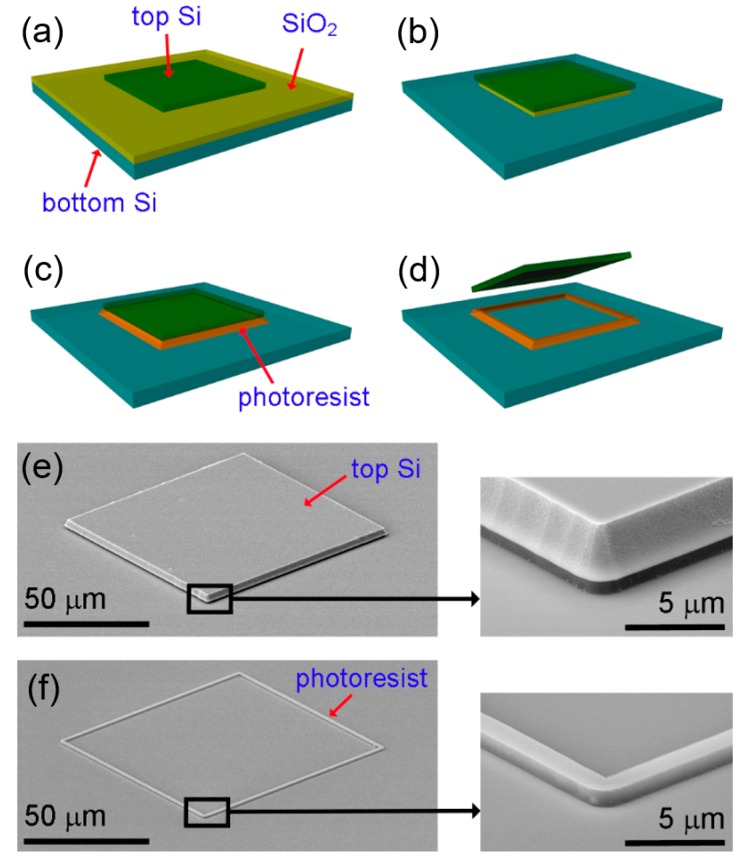
Overview of the process flow to fabricate Si inks which are ready to be retrieved using a stamp from a donor substrate. (**a**) A top Si layer is patterned on a silicon-on-insulator (SOI) wafer. (**b**) A sacrificial layer (SiO_2_) is undercut etched. (**c**) A photoresist is spin-coated on the sample and flood-exposed to UV light. After development, a photoresist under the top Si remains. (**d**) The sacrificial layer is etched away, and the top Si is suspended on the photoresist and ready to be retrieved. Scanning electron microscope (SEM) images of a fabricated Si ink (**e**) and a residual photoresist after the Si ink retrieval (**f**). Reproduced with permission from [34].

Figure 6 depicts the process flow to prepare Si inks from a silicon-on-insulator (SOI) wafer, and shows SEM images of resultant inks [34]. Here, the Si device layer is shaped into Si inks, and the buried oxide layer is used as a sacrificial layer to enable suspended Si inks. The shape of Si inks was determined by patterning a photoresist and then etching the exposed Si device layer using reactive-ion etching (RIE) or deep reactive-ion etching (DRIE). (Figure 6a) Wet etching with hydrofluoric acid (HF) removes the buried oxide layer and creates an undercut trench below the edges of the patterned Si ink. (Figure 6b) Next, a photoresist is spin-coated on the entire wafer and flood-exposed to ultraviolet (UV) light. At this step, only the photoresist under the undercut trench is not exposed to UV light. The wafer is immersed in a developer to remove photoresist everywhere except in the undercut regions (Figure 6c). Finally, the buried oxide layer under the Si ink surrounded by the remaining photoresist is eliminated by HF wet etching [34]. After the final HF etching, the Si ink is suspended and tethered to the underlying Si wafer with the photoresist anchor (Figure 6d). SEM images of a fabricated Si ink and a remaining photoresist anchor structure after the Si ink retrieval are shown in Figure 6e,f. Each image includes a magnified view of the corner in the right inset [34].

Different ink materials may require different sacrificial layers to be prepared as inks. For example, an Si, SiO_2_, or polymethylmethacrylate (PMMA) sacrificial layer is necessary to make SiO_2_, Au, or SU8 ink, respectively [35,59,60,61,62]. However, for any ink materials, inks are photolithographically patterned with desired shapes and entail relatively low adhesion or joining to donor substrates. It is worthwhile to note that the use of photoresist anchors is not necessary when preparing SU8 inks, since shaped SU8 inks on a PMMA sacrificial layer are held on a donor substrate even during PMMA removal in an acetone bath, which is relatively milder than other sacrificial layer etching [35]. Furthermore, composite inks with two or more materials may also be prepared by depositing additional material layers onto the original ink layers during the ink fabrication. For example, Au and graphene were patterned and deposited on Si inks to form composite inks and the metamaterial characteristics of stacked composite inks were demonstrated [63].

## 4. Joining

After transfer printing, inks are placed onto target areas on a receiving substrate relying on van der Waals forces, which are relatively weak. Thus, to ensure the structural integrity of micro-LEGO-assembled structures and devices, post-steps converting weak van der Waals interaction to strong interfacial joining after transfer printing are necessary. Furthermore, other interfacial characteristics, such as electrical contact resistance and thermal conductance at the contact interfaces after these steps, should also be desirable or acceptable. Typical strategies for those steps originate from traditional wafer bonding techniques, which involve thermal processing. Depending on assembled material pairs, diverse joining mechanisms, including fusion [64,65], eutectic [66], and adhesive [67] bonding, were exploited in micro-LEGO. It is noted that typical wafer bonding often requires an external force to mate two wafers during thermal processing, but micro-LEGO does not. The contact area between a microscale solid ink and an underneath surface is quite small, such that the effect of surface curvature and the chance to have any surface defects are relatively low compared with wafer-level bonding. Otherwise, they would hinder efficient material joining during thermal processing without an external force in micro-LEGO. In this section, thermal processing conditions dependent on assembled material pairs and the resulting mechanical joining strength characteristics are briefly summarized. Following this, the electrical contact characteristics after thermal processing will be presented.

### 4.1. Thermal Processing

Thermal processing conditions for material joining with various material pairs among Si, SiO_2_, Au, and SU8 are summarized in Table 2. While thermal processing conditions for material joining in micro-LEGO are adopted from conventional wafer bonding techniques, optimal wafer-scale thermal processing conditions are not necessarily exactly replicated at the microscale when implementing micro-LEGO. These established process conditions may be modified for further optimization.

### 4.2. Mechanical Joining Strength

**Figure 7:**
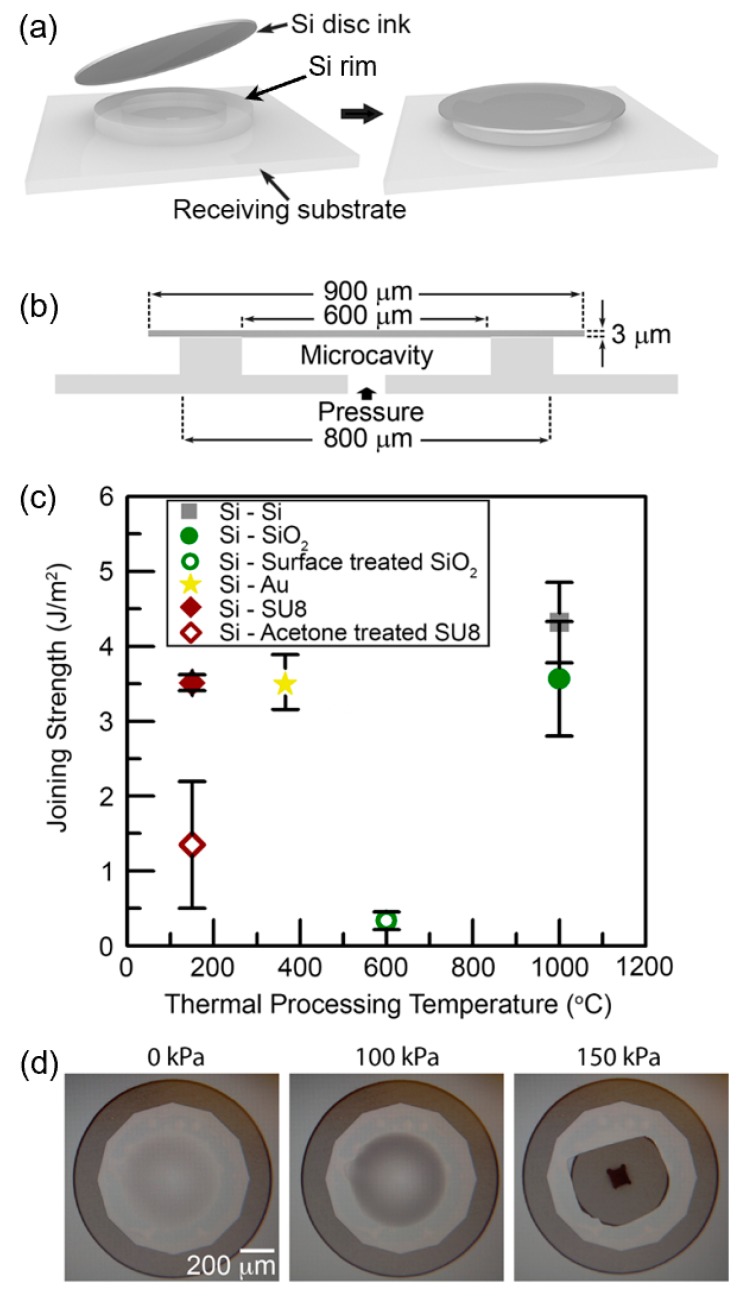
A blister test specimen and results of measured joining strength. (**a**) Schematics of an Si–Si blister test specimen assembled via micro-LEGO. An Si disc ink, separately prepared on a donor substrate, is transferred onto a receiving substrate and joined via thermal processing for hermetic sealing through Si–Si fusion bonding. (**b**) Cross-sectional view of the assembled specimen. (**c**) Joining strength data with respect to material pairs and thermal processing temperatures obtained through blister tests. (**d**) Representative optical microscope images for an Si–Si blister test specimen at three pressure states. Reproduced with permission from [35].

Mechanical joining characteristics between paired materials after thermal processing with the aforementioned conditions were quantitatively investigated [35] adopting a blister test, which was previously utilized to measure the adhesion of thin films formed on Si substrates [68,69]. An Si specimen for a blister test is made based on the schematic procedure and dimensions depicted in Figure 7a,b. When the pressure inside a hermetically sealed microcavity increases in a controlled manner, an Si ink may be delaminated from an Si rim structure on a receiving substrate at critical pressure, which finally determines the joining strength between the Si ink and the Si rim [68]. Based on this procedure, the resultant joining strength data were collected with respect to material pairs and their thermal processing temperatures, as plotted in Figure 7c. Optical images of an assembled Si ink upon pressuring are also shown in Figure 7d, where the ink is ruptured prior to delamination from the underneath Si rim. This ruptured Si ink indicates that the measured critical pressure for an Si–Si pair is associated with the lower bound of the actual joining strength. As summarized in Figure 7c, all obtained data for the four material pairs were similar to or higher than the toughness data for silicon wafer bonding measured elsewhere [64,65,66]. These remarkable results, particularly considering that each joining was achieved without external forces during thermal processing as opposed to wafer bonding techniques, validate the strong structural integrity of MEMS structures or devices that are assembled via micro-LEGO.

### 4.3. Electrical Contact Resistance

**Figure 8:**
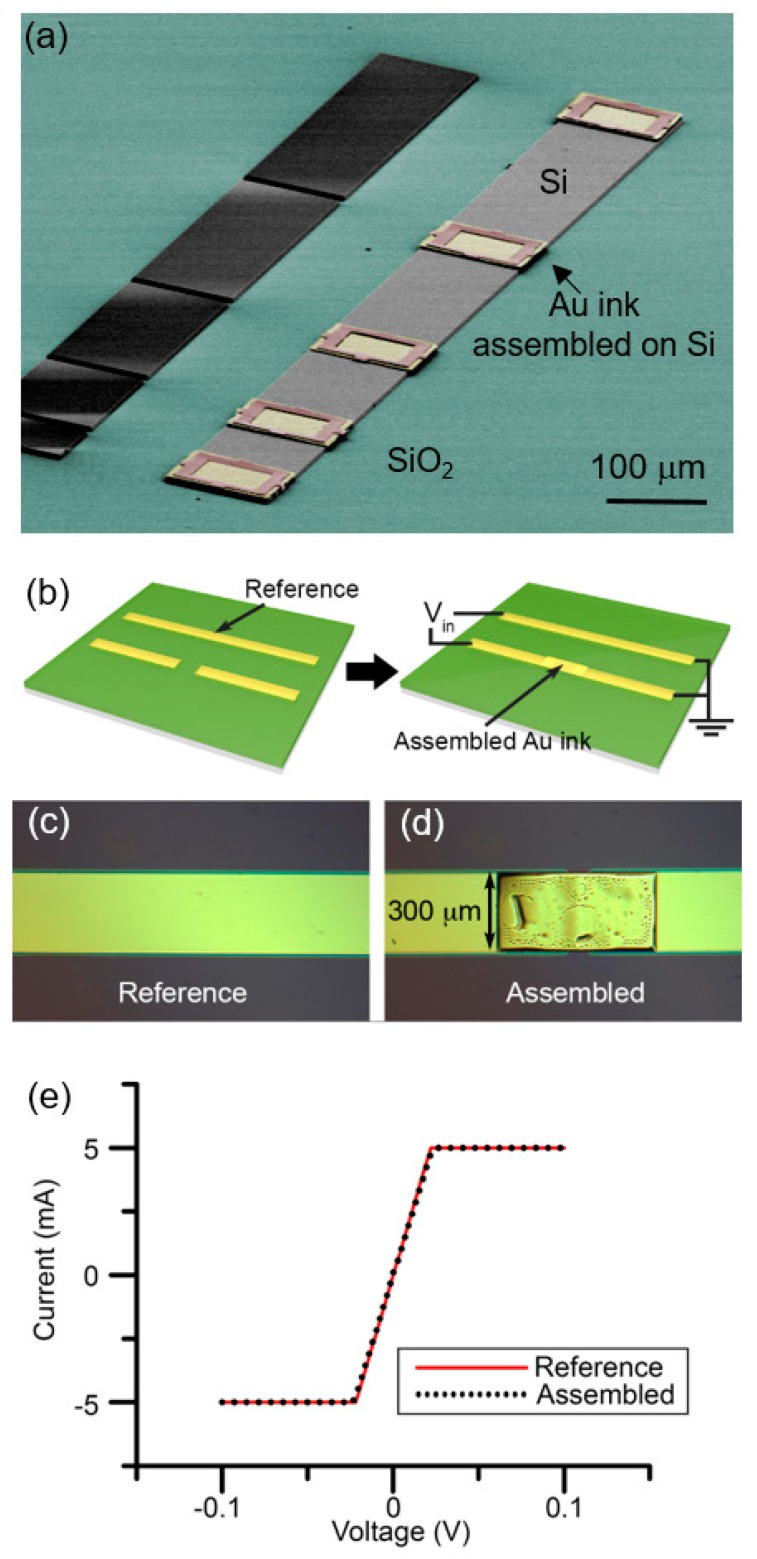
(**a**) A colored SEM image of Au inks (yellow) assembled on an Si strip (grey) which is patterned on SiO_2_ (green) for transmission line model (TLM) measurements. (**b**) Schematics of reference and assembled Au lines. (**c**, **d**) Optical images of a reference Au line fabricated through microfabrication and a connected Au line with an assembled Au ink. (**e**) I–V curves of the reference and assembled Au lines. Reproduced with permission from [35,61].

In order to use micro-LEGO-assembled structures within device applications, the electrical contact at the interface between two assembled materials, especially metal–semiconductor or metal–metal interfaces, should be acceptable. First, a transmission line model (TLM) [70,71,72] was adopted to measure the contact resistance of transfer-printed Au inks on Si (Figure 8a) and their ohmic contact characteristics were demonstrated [61]. It was observed that a transfer-printed Au ink on Si exhibits significantly reduced contact resistance when they are thermally processed at or above the bulk eutectic temperature. However, the surface of Au roughens during high temperature processes, thus, a controlled thermal processing is required to form useful metal–semiconductor contact in micro-LEGO. The change of contact resistance and surface morphology during thermal processing is attributed to the atomic-level mass transport between transfer-printed Au and Si, which results in their eutectic joining and Au dewetting. Next, metal–metal contact formed by micro-LEGO was characterized [35]. An Au ink was assembled on a disconnected Au line via micro-LEGO employing Au–Au cold welding [73] as shown in Figure 8b–d and the current–voltage (I–V) curve was plotted in Figure 8e and compared with that of a reference sample. Given their identical I–V curves, it was demonstrated that assembly of Au inks using micro-LEGO does not result in their altered electrical performance.

## 5. MEMS via Micro-LEGO

Once the process components of micro-LEGO, i.e., transfer printing, ink preparation, and material joining are established, structural and functional microsystems can readily be assembled. In particular, micro-LEGO enables MEMS devices with mechanical and electrical functionalities which are assembled from prepared parts. Thus, the final device architectures can be determined during the assembly with more flexibility to customize device designs. In this section, several examples of fully or partially assembled microsystems are summarized to represent device-level applications of micro-LEGO. First, a variety of microscale structures assembled from Si, SiO_2_, Au, and SU8 are presented. The following examples are microsystems requiring suspended parts, which are assembled without the forming and etching of sacrificial layers. More specific examples of a MEMS scanner and a micromirror demonstrate how micro-LEGO can achieve structural architectures with optical functionalities which are extremely difficult to obtain using conventional microfabrication. Subsequently, microresonators, which also require suspended parts, are shown. Finally, the atomic force microscopy (AFM) with improved performance due to special components being assembled on conventional AFM cantilevers is introduced.

### 5.1. 3D Heterogeneous Microstructures

**Figure 9:**
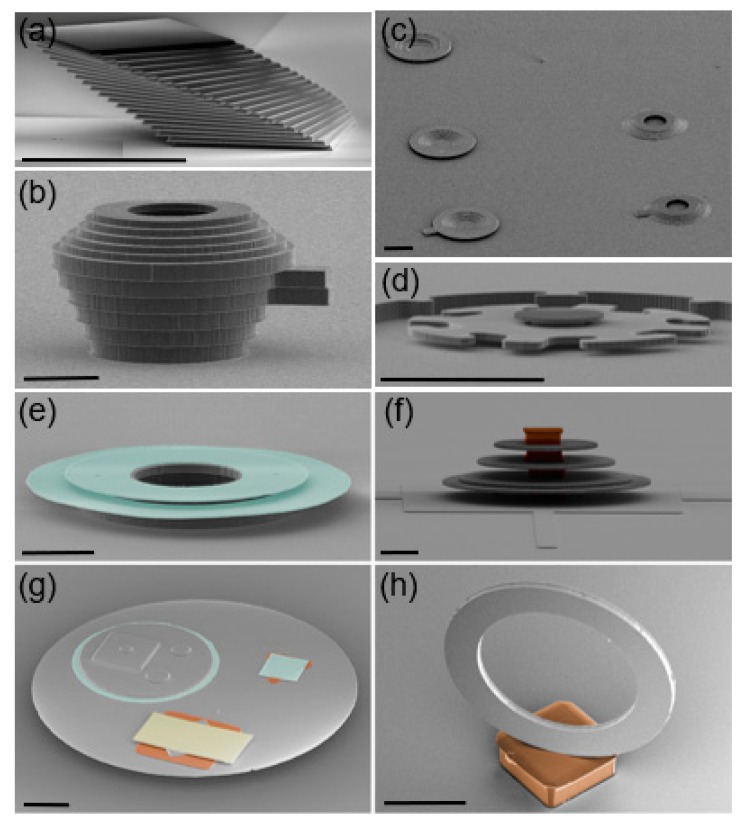
Colored SEM images of micro-LEGO-assembled Si (grey), SiO_2_ (green), Au (yellow), and SU8 (brown). Images of (**a**) multilayer configurations of thin Si platelets in a single stack with small incremental rotations and translations, (**b**) stacked thick Si rings with varied thickness and diameter, (**c**) stacked thin Si rings with varied diameter, (**d**) an assembled micromotor representation, (**e**) double layer Si rings and SiO_2_ discs, (**g**) a microstructure composed of Si, SiO_2_, Au, and SU8 pieces, (**f**) a multiple layer Si discs and SU8 blocks, and (**h**) a vertically aligned Si ring joined on a SU8 block. All scale bars represent 100 m. Reproduced with permission from [31,34,35,53].

3D heterogeneous microstructures which are assembled via micro-LEGO have been extensively demonstrated over the past several years. Figure 9 provides colored SEM images of various 3D microstructures assembled with silicon (Si, grey), silicon dioxide (SiO_2_, green), gold (Au, yellow), and epoxy resist (SU8, brown) inks. Here, different colors represent different ink materials. A multilayer configuration of 100 μm wide and 3 μm-thick Si square inks in a single stack with translational and rotational increments is shown in Figure 9a [31]. 10 and 50 μm-thick Si inks were prepared and stacked to represent a teapot-like microstructure (Figure 9b) [34]. This teapot-like structure was modified with 3 μm-thick Si ring-shaped inks to be upturned and downturned dish-like microstructures (Figure 9c) [34]. Figure 9d shows an assembled microstructure mimicking a micromotor which consists of three separately fabricated and assembled Si parts, i.e., a substrate consisting of a stator and a rotor-axle, a rotor, and a cap which is assembled on the top of the rotor-axle to constrain the rotor in place [53]. This simplified stator design does not allow the motor’s actuation by electrostatic force as other existing examples, but its freedom to rotate about its axle is maintained as demonstrated in [53]. While these example microstructures are composed of Si inks, other examples shown in Figure 9e–h depict 3D heterogeneous materials integration [35]. This material heterogeneity is possible since the protocols to prepare inks with other materials and to achieve their joining were additionally established. A microstructure with double layer Si rings and SiO_2_ discs (Figure 9e) and the other with multilayer Si discs and SU8 blocks (Figure 9f) provide the integration between semiconductors and dielectric materials via micro-LEGO. Figure 9g highlights the heterogeneous integration of four disparate classes of materials, i.e., semiconductor (Si), dielectric (SiO_2_), metal (Au), and polymer (SU8). A vertically aligned Si ring on a SU8 block in Figure 9h is also possible if the orientation of the Si ring ink is changed on a stamp before it is printed [35]. While the 3D heterogeneous microstructures shown in Figure 9 do not provide device functionalities, these microstructures assembled from diverse inks at the microscale present the unparalleled 3D heterogeneous material assembly capabilities of micro-LEGO that can further be exploited in numerous applications.

### 5.2. Out-Of-Plane Vertical Comb Drive

**Figure 10:**
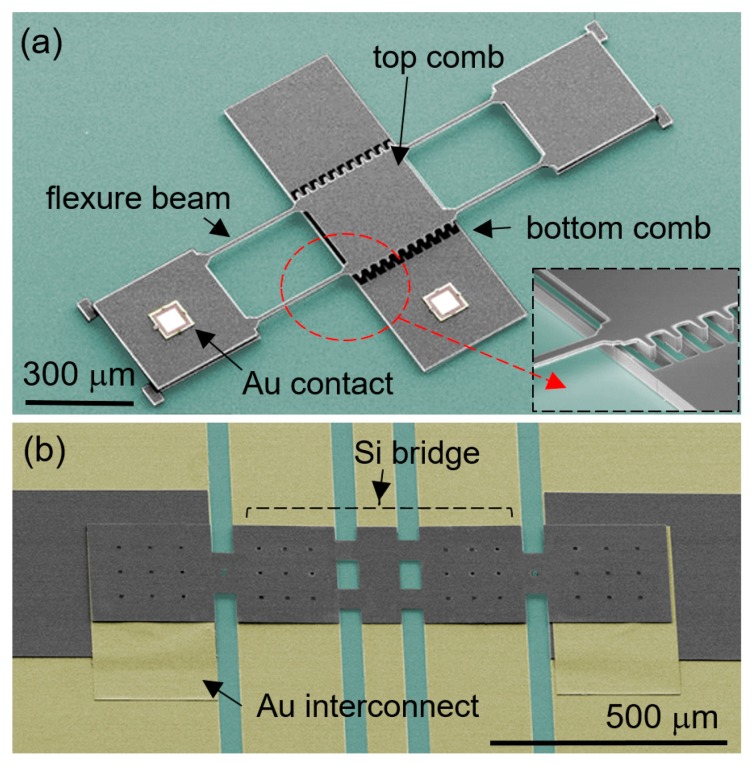
Colored SEM images of (**a**) a microelectromechanical systems (MEMS) vertical comb drive and (**b**) an RF MEMS switch assembled with Si (grey) and Au (yellow) parts on SiO_2_ (green) surfaces. Reproduced with permission from [35,79].

A comb drive is a type of dominating sensing and actuating MEMS devices which commonly entails in-plane or horizontal motion of in-plane combs, mainly because a comb drive is made through monolithic microfabrication including a sacrificial layer etching step. Therefore, a comb drive with out-of-plane or vertical motion is inherently more complex to design and fabricate [74,75,76,77]. A straightforward way to produce a vertical comb drive bypassing the complex fabrication procedure is to vertically align and assemble prefabricated combs together. To this end, a hidden vertical comb drive actuator was first assembled on a PDMS substrate by ‘part-transfer’ [78]. More recently, a vertical comb drive with out-of-plane comb teeth was assembled on an Si substrate via micro-LEGO, as shown in Figure 10a [79]. This out-of-plane vertical comb drive includes two Au contact pads which are also assembled via micro-LEGO. The characterized sensing and actuation capabilities of the comb drive proposed further additional opportunities for use in devices, such as microscale weight sensors, micromirrors, and vibration energy harvesters.

### 5.3. RF MEMS Switch

Radio frequency (RF) MEMS switch is another device class that often needs a suspended part. Micro-LEGO was also exploited to build a series contact-type RF MEMS switch. The suspended part of a common RF MEMS switch for its electromechanical performances is complex to produce using microfabrication, due to limited material choice for a sacrificial layer as well as stiction during wet processes [80]. Micro-LEGO alleviated these manufacturing challenges since it consecutively transfers and joins individual components of a device in dry conditions. Therefore, it significantly simplified the manufacturing procedure and provided more flexibility in device design than microfabrication and other transfer techniques [81,82]. Figure 10b represents a colored SEM image of the RF MEMS switch fully assembled via micro-LEGO [35]. To characterize its device functionalities, voltage bias was applied between a suspended beam and two ground lines on each side of the center signal line. Upon voltage bias, the suspended beam deflects down via the electrostatic force. In open state, the resultant data showed high insertion loss similar to that of a coplanar waveguide (CPW) substrate as the center signal line was disconnected. The insertion loss is, however, significantly reduced when biased, since the fully deflecting beam makes physical contact with the disconnected signal line and induces electrical connection. While this experimentally measured insertion loss change of the RF MEMS switch exhibited its device functionalities, its performance is not on par with that of other state-of-the-art devices. With more optimized designs and processes, micro-LEGO potentially enables the development of RF MEMS switches with improved performances.

### 5.4. Two-Axis MEMS Scanner with Photonic Crystal Mirrors

**Figure 11:**
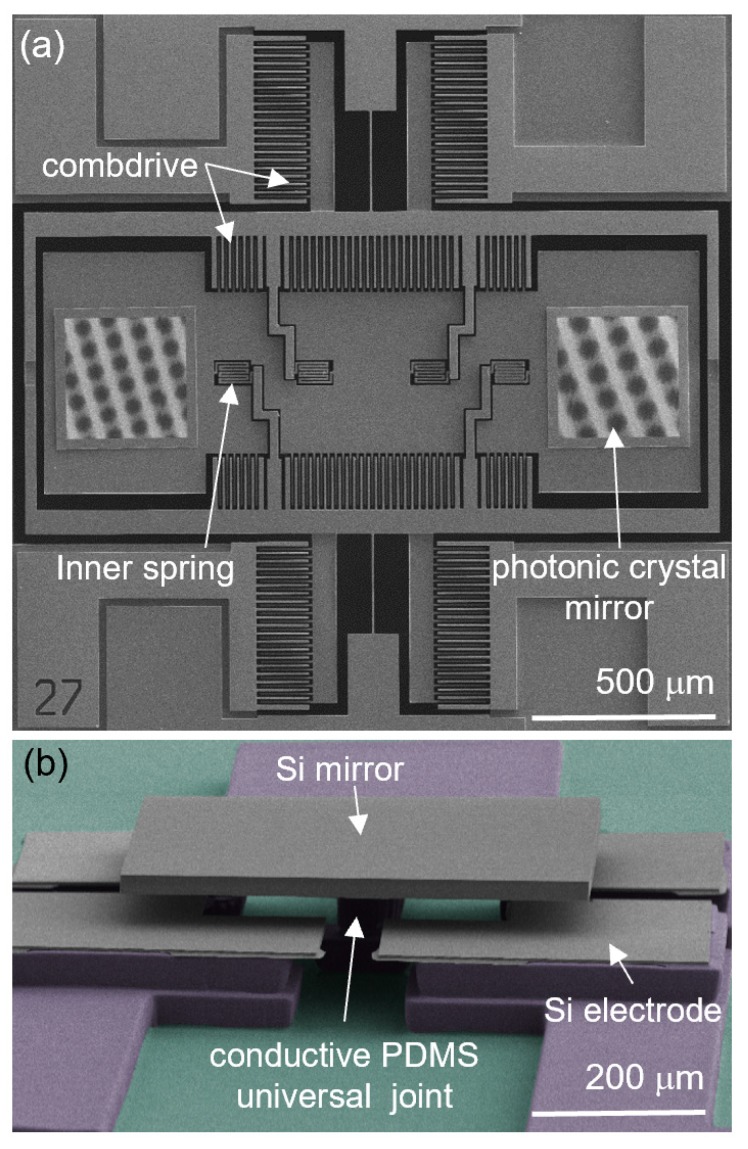
Colored SEM images of (**a**) a MEMS scanner with assembled photonic crystal mirrors and (**b**) a micromirror with a highly doped Si (grey) mirror on a conductive PDMS universal joint (purple). Reproduced with permission from [95,96].

The use of photonic crystal mirrors [83,84,85,86,87] are promising in applications such as 3D displays using laser-induced breakdown plasma [88], high power laser beam steering [89], and coherent communications [90], due to their many unique features. Photonic crystal mirrors, which are formed by periodic patterning of high-refractive-index films, maintain the high reflectivity and robustness of Bragg mirrors. The reflection spectrum of photonic crystal mirrors is altered by manipulating the hole size, pitch, and slab thickness from visible to infrared (IR) wavelengths [91]. Thus far, high-reflectivity MEMS scanners with photonic crystal mirrors have been fabricated by forming photonic crystal mirrors directly onto the surface of MEMS scanners [92,93], increasing the complexity of the fabrication process. To reduce the fabrication complexity, micro-LEGO was exploited and a two-axis MEMS scanner with assembled high-reflectivity broadband photonic crystal mirrors was fabricated as displayed in Figure 11a. Stress-free, monolithic photonic crystal mirrors were formed in a single crystal silicon (SCS) device layer of a SOI wafer, which were further processed to be inks [94]. Then, these photonic crystal mirror inks were assembled on a MEMS scanner using transfer printing and thermal processing [95]. In this example, micro-LEGO provided more design flexibility since optical components with disparate characteristics can be integrated onto a common MEMS platform. The photonic crystal mirrors show higher than 85% reflectivity from 1490 nm to 1505 nm wavelength, and higher than 90% reflectivity from 1550 nm to 1570 nm wavelength [95].

### 5.5. Tip–Tilt–Piston Micromirror with an Elastomer Universal Joint

After the previously described MEMS scanner where the actuation parts are made via microfabrication, a hybrid tip–tilt–piston micromirror shown in Figure 11b was fully assembled via micro-LEGO [96]. Predominant MEMS-based micromirror designs retain a gimbaled structure which allows two-axis motion. However, there are limitations of a gimbaled structure, including large footprint and unequal frequency responses with respect to the two axes [74,95]. Accordingly, gimbal-less structures with two-axes motion were studied, but they usually require even more complex design and fabrication processes [97,98]. To tackle these challenges, micro-LEGO was utilized and a gimbal-less hybrid tip–tilt–piston micromirror consisting of an elastomer universal joint was built with even less fabrication complexity. The micromirror was composed of a highly doped SCS mirror and a conductive elastomer universal joint, which were mechanically joined and electrically connected. This unique device design, enabled by micro-LEGO, benefits from combining two distinct materials to achieve a high-quality reflective surface using SCS as well as a highly flexible universal joint using elastomer. To realize this hybrid system, micro-LEGO protocols were extended such that a SCS mirror and conductive carbon black-embedded elastomer parts were prefabricated separately and integrated afterward. Here, the elastomer parts were partially cured before transfer printing of the SCS mirror to enhance their adhesion. This was followed by low temperature (60 °C) thermal processing to fully cure the elastomer part and to create a strong covalent bond with SCS via a hydroxyl condensation reaction [99]. In such a way, the elastomer components do not need to experience harsh process environments, including high temperature and, therefore, a strong mechanical and electrical connection between two heterogeneous materials was achieved without any damage to the elastomer. The static and dynamic characteristics of the micromirror were tested and the identical response of its two orthogonal scanning axes was measured. Resonant frequencies for x- and y-axis rotations were determined to be 1.2 kHz, which match the values estimated by modal analysis [96]. The quality factors for both axes were also determined to be 2.1 [96]. In addition, the piston stroke by compressive deformation of the elastomer universal joint along the z-axis was quantified.

### 5.6. Nanoplate Resonators

**Figure 12:**
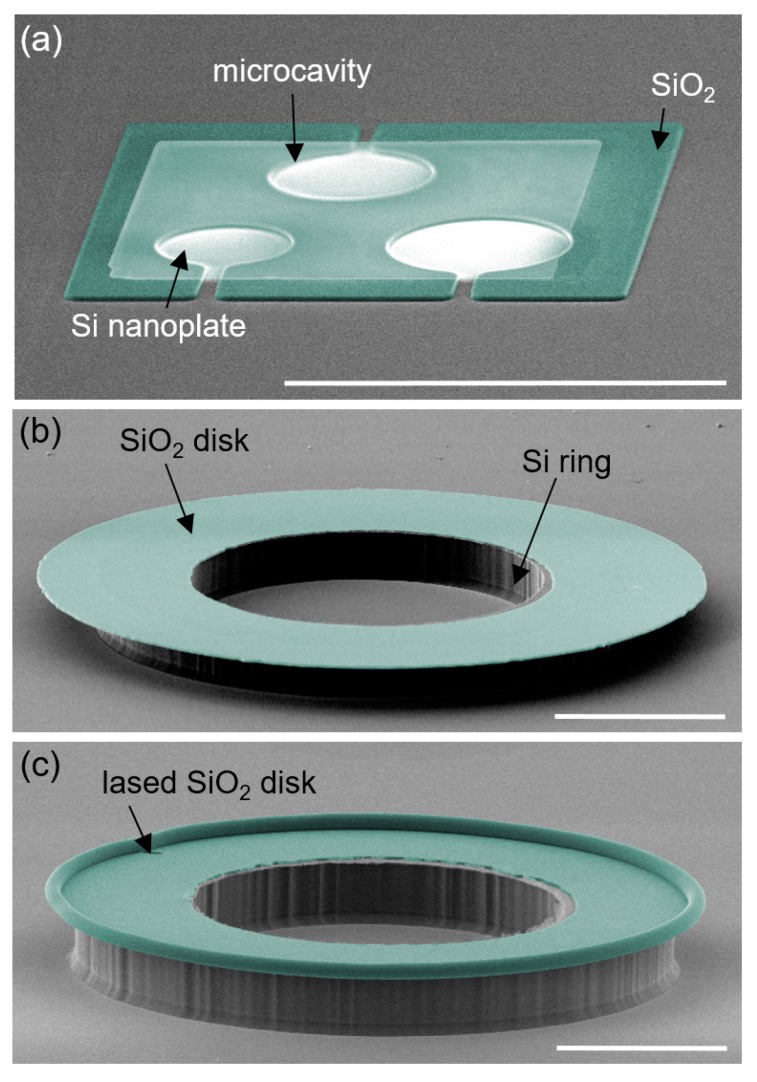
(**a**) A colored SEM image of a nanoplate resonating structure with an Si nanoplate assembled on SiO_2_ support. Colored SEM images of (**b**) an SiO_2_ disk assembled on an Si ring and (**c**) a microtoroid resonator after lasing SiO_2_ disk. All scale bars represent 100 μm. Reproduced with permission from [35].

Using nanoscale plate resonators instead of common cantilever-based resonators exhibits several advantages, such as higher quality factor (Q-factor) for an equivalent mass and more structural robustness for mass sensing in fluid [100,101,102,103,104]. However, enabling such a nanoscale plate, which is suspended, has relied on the selective etching of a sacrificial layer, which is limited regarding materials and geometries, due to potential stiction issues. Micro-LEGO bypassed these limitations and was used to assemble nanoplate resonators composed of 340 nm-thick SCS plates on 1 m-thick SiO_2_ bases which had multiple microcavities, as displayed in Figure 12a. The structural integrity of nanoplates ensured by micro-LEGO allowed the fabrication of multiple suspended nanoplates in a single step and on the same single base, without mechanical crosstalk between them [105]. After the structural design was further modified and additional interconnects were formed, the nanoplate resonators performed with integrated electrostatic actuation and capacitance-sensing capabilities [106]. Those nanoplate resonators were tested by measuring their resonant frequency in a fully integrated manner. The results indicated that they performed as predicted by theory, and provided quality factors of more than 30 in air [106].

### 5.7. Microtoroid Resonators

The assembly of a microtoroid-shaped photonic whispering-gallery resonator (WGR) is another example to build through micro-LEGO with less fabrication complexity [35]. WGRs were extensively fabricated and explored in nonlinear optics due to their extremely high optical Q-factors [107,108,109,110]. In particular, ultra-high-Q silica (SiO_2_) microtoroid resonators are typically fabricated by chemical etching and laser reflow. Specifically, the fabrication processes include Xe_2_F isotropic etching of an Si substrate to form an undercut silica disk, which becomes a microtoroid by physical reflow using high power CO_2_ laser illumination [111]. This conventional approach involves the challenge that an ultra-high-Q silica resonator cannot be co-integrated with other planar photonic and electronic devices, since the resulting Si substrate is nonplanar and often pitted. On the other hand, micro-LEGO can realize this geometry with no need for undercut, as it assembles prefabricated Si and SiO_2_ ring-and disk-shaped inks individually. Furthermore, micro-LEGO allows for more complex multilayered WGR geometries (Figure 9e) which cannot be accommodated by conventional microfabrication processes. Figure 12b,c shows SEM images of the device before and after reflow of the SiO_2_ disk. Tapered fiber-coupling [112] was adopted to test this assembled microtoroid resonator. A characteristic Lorentzian-shaped 0.087 nm-wide optical resonance of the WGR at 1549 nm and extracted Q-factor of about 1.7 × 10^4^ were measured by reading the optical transmission through the waveguide [35].

### 5.8. Atomic Force Microscopy Using an Inner-Paddled Cantilever

**Figure 13:**
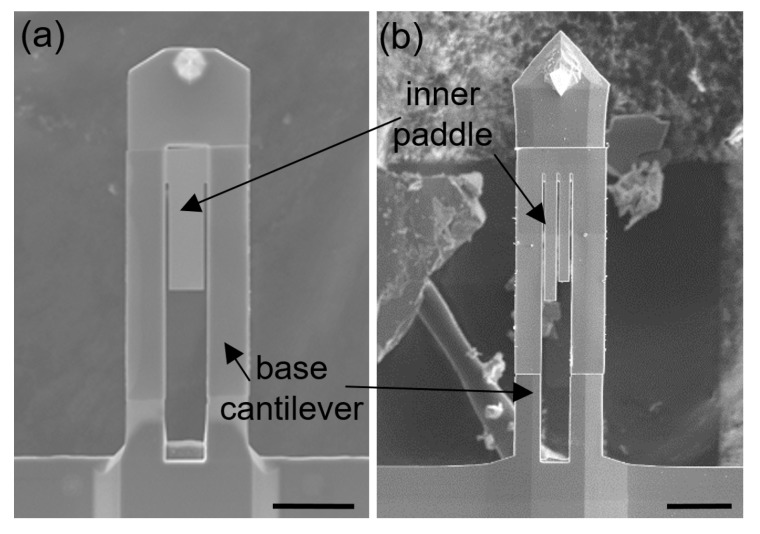
SEM images of AFM cantilevers with single (**a**) and double (**b**) inner paddles which are formed by micro-LEGO and focused ion beam (FIB) milling. Both scale bars represent 20 m. Reproduced with permission from [116].

Atomic force microscopy (AFM) has been widely used to explore topographical features and local mechanical properties of material samples with subnanometer spatial resolution [113,114]. Recently, a noble AFM probe design of inner-paddled cantilever was proposed, which resolved part of the challenges associated with conventional AFM probes, such as the low signal-to-noise ratio of higher harmonic signals and the crosstalk between the observables. Micro-LEGO was successfully adopted to enable the inner-paddled cantilever design. This design involves an inner paddle which is much thinner than an AFM cantilever where it is rigidly fixed. Since building a thin paddle inside a relatively thick cantilever is quite difficult using traditional monolithic microfabrication, it was implemented using a combination of processes, including micro-LEGO. The typical procedure is composed of a few steps [115]. After a rectangular cavity is carved out on a regular AFM cantilever with a focused ion beam (FIB), an Si membrane with a desired thickness is assembled onto the cantilever via micro-LEGO. The Si membrane is subsequently carved out in a single or multiple inner paddle shape with FIB, as shown in Figure 13. The fabrication procedure is quite simple but powerful, allowing for this specific inner-paddled AFM probe design. Using this AFM probe design, intentional nonlinear internal resonance was demonstrated for the enhancement of higher harmonics, leading to improved simultaneous topography imaging and compositional mapping [116]. More recently, the efficacy of this AFM probe design was also demonstrated via piezoresponse force microscopy (PFM) [117].

## 6. Conclusions and Future Outlook

In this review, a recently established microassembly, i.e., micro-LEGO, and several examples of MEMS applications enabled by micro-LEGO were presented. Micro-LEGO involves transfer printing and thermal processing for the transfer and joining of desired materials, including silicon, silicon dioxide, gold, and SU8, representing semiconductor, dielectric, metal, and polymer materials, respectively. Thus far, studies have demonstrated that micro-LEGO enables high structural integrity and sound electrical contact properties of assembled structures for device-level applications. Therefore, this newly developed microassembly strategy reduces the fabrication process complexity and simplifies the entire fabrication procedure to achieve various 3D shape structures and devices. Whereas micro-LEGO provides high potentials to allow diverse MEMS applications as demonstrated, there are several challenges to overcome, such as difficulty of batch processing, moderate process yield, thermal processing of the entire system, and limited material choices. Those challenges must be addressed in future, in order to allow micro-LEGO to be more broadly adopted in MEMS in the context of process scalability as well as flexibility. For example, more precise parallel transfer printing in a more controlled environment would allow higher process throughput and yield. Laser-assisted local thermal processing may enable individual material joining on a single substrate. While there are fundamental and technical difficulties with regard to drastically expanding the material choices that can be assembled via thermal processing in micro-LEGO, assembling material candidates will be further explored. Despite these current challenges, the outlook for micro-LEGO for MEMS is promising, since it complements other existing fabrication processes to make the manufacture of traditionally unachievable device architectures possible. More efforts on improving transfer printing and developing protocols for ink preparation and thermal processing with diverse materials should continuously be made, to discover more applications of micro-LEGO in MEMS and other related areas.

## Figures and Tables

**Table 1:** Comparison of adhesive strength and reversibility for various stamps available in the literature [33].

Material	Stamp Surface Geometry	Max Adhesion (kPa)	Reversibility (max/min)	Adhesion Control	References
PDMS	Flat	50	50:1	inflation	[43]
		85	>10:1	shear motion	[44]
		150	3:1	kinetic	[31]
	Flat (angled side walls)	100	100:1	shear motion	[45]
	Microtip	80	>1000:1	contact area change	[31]
	Pedestal	1600	2:1	kinetic	[55]
			∞:1	laser-heating	[55]
ST-1087	Flat	1450	39:1	buckling	[46]
SMP	Flat	3200	6:1	rigidity change	[33,53]
	Microtip	2800	>1000:1	rigidity & contact area change	

**Table 2:** Common material joining conditions implemented in micro-LEGO [35].

Receiving Material	Ink Material	Joining Condition
Si	Si	An Si ink is directly transfer-printed onto a target Si surface and thermally processed in a furnace at 1000 °C for 10 min with 5 s ramping.
Si	SiO_2_	A SiO_2_ ink is directly printed onto an Si surface and thermally processed in a furnace at 1000 °C for 10 min with 5 s ramping.
Si	Au	The surface of Si is cleaned with HF for removal of native oxide layer followed by transfer printing of an Au ink. The Au ink is transfer-printed within short period of time after the HF treatment. The transfer-printed sample is then placed in a furnace and thermally processed at 365 °C for 10 min with 5 s ramping.
Si	SU8	An SU8 ink is directly printed on an Si surface and thermally processed in a furnace at 150 °C for 10 min with 10 min ramping.
SiO_2_	Si	A Si ink is directly printed onto an SiO_2_ surface and thermally processed in a furnace at 1000 °C for 10 min with 5 s ramping.
Au	Au	An Au ink is printed onto a clean Au surface with a moderate pressuring for more intimate contact.
SU8	Si, Au, SiO_2_	A desired ink is printed and thermally processed in a furnace at 150 °C for 10 min with 10 min ramping.
